# The habenula-targeting neurons in the mouse entopeduncular nucleus contain not only somatostatin-positive neurons but also nitric oxide synthase-positive neurons

**DOI:** 10.1007/s00429-021-02264-1

**Published:** 2021-03-31

**Authors:** Yuta Miyamoto, Takaichi Fukuda

**Affiliations:** Department of Anatomy and Neurobiology, Graduate School of Medical Sciences, Kumamoto University, Honjo 1–1-1, Chuo-ku, Kumamoto, 860-8556 Japan

**Keywords:** Basal ganglia, Immunohistochemistry, Connectivity, Fluoro-gold

## Abstract

The entopeduncular nucleus (EPN) in rodents is one of the two major output nuclei of the basal ganglia and corresponds to the internal segment of the globus pallidus in primates. Previous studies have shown that the EPN contains three types of neurons that project to different targets, namely, parvalbumin (PV)-, somatostatin (SOM)-, and choline acetyltransferase-positive neurons. However, we have recently reported that neurons lacking immunoreactivities for these substances are present in the EPN. Here, we demonstrate that 27.7% of all EPN neurons showed immunoreactivity for nitric oxide synthase (NOS). Among them, NOS-only positive and NOS/SOM double-positive neurons accounted for 20.1% and 6.8%, respectively, whereas NOS/PV double-positive neurons were rarely observed. NOS-containing neurons were distributed in a shell region surrounding the thalamus-targeting, PV-rich core region of the EPN, especially in the ventromedial part of the shell. The retrograde tracer fluoro-gold (FG) was injected into several target regions of EPN neurons. Among FG-labeled EPN neurons after injection into the lateral habenula (LHb), NOS-only positive, NOS/SOM double-positive, and SOM-only positive neurons accounted for 25.7%, 15.2%, and 59.1%, respectively. We conclude that NOS-positive neurons are the second major population of LHb-targeting EPN neurons, suggesting their possible involvement in behaviors in response to aversive stimuli.

## Introduction

An influential concept of the neural circuits in the basal ganglia is expressed as the direct/indirect pathway scheme (Albin et al. 1989; Alexander and Crutcher 1990), to which another important pathway, called hyperdirect, has been added (Nambu et al. 2000, 2002). The rodent entopeduncular nucleus (EPN) is one of the output nuclei of these pathways and corresponds to the internal segment of the globus pallidus (GPi) in primates. The main targets of the EPN/GPi are the ventral anterior-ventral lateral thalamus (VA-VL), the parafasicular-center median complex (PF-CM), the pedunculo-pontine tegmental nucleus (PPN) of the brainstem, and the lateral habenula (LHb) (Nauta and Mehler 1966; van der Kooy and Carter 1981; Parent and De Bellefeuille 1982; Ilinsky and Kultas-Ilinsky 1987; Parent et al. 1999). These areas are targeted in a separable manner by two types of EPN/GPi neurons with different morphological characteristics (Kha et al. 2000; Parent et al. 2001). GABAergic EPN neurons containing the calcium-binding protein parvalbumin (PV) target the VA-VL, PF-CM, and PPN (Rajakumar et al. 1994), whereas somatostatin (SOM)-containing neurons project to the LHb (Vincent and Brown 1986). Recently, the projection to the LHb has gained much attention, because it is critically involved in the evaluation of action outcomes and the avoidance of aversive stimuli (Matsumoto and Hikosaka 2007; Proulx et al. 2014; Stephenson-Jones et al. 2016), a mechanism antagonistic to the well-established circuitry for reinforcement learning through activation of midbrain dopaminergic neurons.

However, previous morphological studies have shown that EPN/GPi neurons are more heterogeneous than this simplified scheme. For example, choline acetyltransferase (ChAT)-positive neurons in rodents are present in the rostral (Moriizumi and Hattori 1992) and peripheral (Miyamoto and Fukuda 2015) parts of the EPN. The rostral ChAT-positive neurons project mainly to the frontal cerebral cortex with some axonal collaterals to the LHb (Moriizumi and Hattori 1992). Moreover, electrophysiological (Li et al. 2019) and genetic (Wallace et al. 2017) studies have also reported multiple types of neurons other than SOM neurons in the EPN–LHb pathway, although this population remains to be characterized morphologically.

In our previous study, we demonstrated the presence of EPN neurons that did not show immunoreactivity for any of PV, SOM, or ChAT in the mouse (Miyamoto and Fukuda 2015). The proportion of these neurons to all EPN neurons was as high as 20%. Therefore, we analyzed the immunohistochemical properties and distribution pattern of this unidentified population of EPN neurons and further clarified their projection targets by tracer injection experiments.

## Materials and methods

### Fixation and tissue preparation

All experiments were performed according to the Guide for the Care and Use of Laboratory Animals (National Institutes of Health Publications No. 80-23, revised 1996), and all protocols were approved by the Institutional Animal Care and Use Committee at Kumamoto University. In this study, every effort was made to minimize the number of animals used and their suffering.

Fifteen male C57BL/6 J mice (20–26 g, 7–9 weeks old) were deeply anesthetized by inhalation of isoflurane and were perfused via the ascending aorta with 0.01 M phosphate-buffered saline (PBS, pH 7.4) followed by 50 ml of 4% paraformaldehyde (PFA) in 0.1 M phosphate buffer (PB, pH 7.4) at room temperature. Brains fixed with PFA were removed from the skull and stored overnight in the same fixative at 4 °C. The next day, the fixative was replaced with PBS containing 0.1% sodium azide.

### Injection of retrograde tracers

Twelve mice were used for the tracer injection experiment. The retrograde tracers used were fluoro-gold (FG, Fluorochrome, 526–94,003) dissolved in saline to a final concentration of 4%. FG was injected stereotaxically into the LHb (AP: − 1.82 mm, ML: 0.4 mm, DV: 2.4 mm, *n* = 3 animals), VA-VL (AP: − 0.95 mm, ML: 1.0 mm, DV: 3.25 mm, *n* = 3 animals, or AP: − 1.34 mm, ML: 1.5 mm, DV: 4.2 mm, *n* = 3 animals), and frontal associational cortex (FrA, AP: 2.77 mm, ML: 1.5 mm, DV: 0.4 mm, *n* = 3 animals) under anesthesia by inhalation of 0.5–2.0% isoflurane using a stereotaxic frame (SR-5 M-HT, Narishige Scientific Instrument Lab). Then, a burr hole was drilled in the appropriate position of the skull, and a glass microelectrode (outside tip diameter 40 µm) containing tracer solution was inserted into the brain. FG was injected iontophoretically into targeted sites by passing a positive-pulsed 5–7 µA duty cycle (2 s on/2 s off). After surgery, wound was closed and topical analgesic (2% Lidocaine gel, Fujisawa) was applied to the wound. Mice were housed singly in small compartments that were temperature- (20 °C) and light-controlled (12 h light/12 h dark cycle). After a survival period of 7 days, the mice were perfusion-fixed as described above.

### Immunohistochemistry

Serial 40-µm-thick coronal sections were cut using a vibrating microtome (TTK-3000, Dosaka) from brain blocks containing the entire EPN. After cryoprotection in 25% sucrose in PBS, sections placed on aluminum foil were rapidly frozen in liquid N_2_ vapor, rapidly thawed in 25% sucrose in PBS, and then processed for triple-fluorescent immunohistochemistry, as previously described (Fukuda and Kosaka 2000; Miyamoto et al. 2019), using slight modifications. The primary and secondary antibodies used in this study are listed in Tables 1 and 2, respectively. Briefly, the first set of sections was incubated in blocking solution containing 5% normal donkey serum (Jackson ImmunoResearch), 0.3% Triton-X and 0.1% sodium azide in PBS overnight, followed by a mixture of rat anti-SOM (1:250, Millipore), rabbit anti-PV (1:5,000, Swant), and sheep anti-NOS (1:10,000, a gift from Dr. Emson) antibodies dissolved in the above blocking solution for 7 days at 20 °C, with a mixture of Alexa 488-conjugated donkey anti-rat IgG (1:250, Jackson ImmunoResearch), Alexa 647-conjugated donkey anti-rabbit IgG (1:250, Jackson ImmunoResearch), and rhodamine red (RR)-conjugated donkey anti-goat IgG (1:250, Jackson ImmunoResearch) overnight. The long incubation period with primary antibodies was essential to improve permeation of the antibodies into the deep portions of the 40-µm-thick sections to obtain confocal images of consistent and sufficient quality throughout the depth of the sections (Fukuda et al. 1998; Fukuda and Kosaka 2000). The second set of immunostaining was performed using a mixture of rat anti-substance P (SP) (1:500, Millipore), mouse anti-NeuN (1:500, Millipore), and sheep anti-NOS antibodies for 7 days at 20 °C, with biotinylated donkey anti-rat IgG (1:250, Jackson Immunoresearch) overnight, and with a mixture of Alexa 488-conjugated donkey anti-mouse IgG (1:250, Jackson ImmunoResearch), streptavidin-Alexa 647 (1:250, Jackson ImmunoResearch), and RR-conjugated donkey anti-goat IgG overnight. Other sets of immunostaining were applied to sections prepared from mice injected with FG into the LHb, VA-VL, and FrA. Triple immunostaining of these sets was performed using a rabbit anti-FG antibody (1:2,500, Millipore) plus a mixture of antibodies recognizing different subpopulations of EPN neurons, as shown in Tables 1 and 2.

**Table 1:** Antibody host species dilution source cat. #/ lot # RRID

Antibody	Host species	Dilution	Source	cat. #/ lot #	RRID
SP	Rat	1:500	Millipore	MAB356/ 3,186,092	AB_94639
SOM	Rat	1:250	Millipore	MAB354/ 3,059,611	AB_2255365
PV	Mouse	1:5000	Swant	235/ 10–11 (F)	AB_10000343
PV	Rabbit	1:5000	Swant	PV25/ 5.10	AB_10000344
NOS	Sheep	1:10,000	Gift from Dr. Emson		AB_2314957
NeuN	Mouse	1:500	Millipore	MAB377/ 2,592,741	AB_2298772
NeuN	Guinea pig	1:1000	Millipore	ABN90P/ 3,107,382	AB_2341095
ChAT	Goat	1:1000	Millipore	AB144P/NG1915294	AB_2079751
FG	Rabbit	1:2500	Millipore	AB153-l/2,668,490	AB_2632408

**Table 2:** Antibody and fluorochrome fluorophore dilution source code. #/ lot. # RRIDTba

Antibody and fluorochrome	Fluorophore	Dilution	Source	code. #/ lot. #	RRID
Donkey anti-rat IgG	Biotin	1:250	Jackson	712–065-153/ 138,378	AB_2315779
Donkey anti-goat IgG	Biotin	1:250	Jackson	705–065-147/ 129,472	AB_2340397
Donkey anti-rat IgG	Alexa488	1:250	Jackson	712–545-150/ 131,953	AB_2340683
Donkey anti-mouse IgG	Alexa 488	1:250	Jackson	715–545-151/ 135,209	AB_2341099
Donkey anti-rabbit IgG	Alexa 488	1:250	Invitrogen	A21206	AB_2535792
Donkey anti-rabbit IgG	Alexa 647	1:250	Jackson	711–605-152/ 1,383,147	AB_2492288
Donkey anti-goat IgG	Rhodamine red	1:500	Jackson	705–295-147/ 140,849	AB_2340423
Donkey anti-mouse IgG	Rhodamine red	1:500	Jackson	715–295-150/97,212	AB_2340831
Donkey anti-guinea Pig	Cy3	1:250	Jackson	706–165-148/ 119,998	AB_2340460
Streptavidin	Alexa 647	1:250	Jackson	016–600-084/ 135,095	AB_2341101

Sections were mounted in Vectashield (Vector Laboratories) and examined using a confocal laser-scanning light microscope (C2, Nikon), which was equipped with three single laser beams, 488, 543, and 633 nm in wavelength, and a filter set of BA 515/30, BA 590/50, and 650 LP. Control sections were prepared by omitting primary antibodies and by mismatching secondary antibodies. Both sets of controls provided only weak, nonspecific staining.

### Confocal laser scanning microscopy

All images for confocal laser scanning light microscopy (CLSM) were obtained using 4 × (Plan Apo, N.A. = 0.2, Nikon), 10 × (Plan Fluor, N.A. = 0.3, Nikon), and 20 × (Plan Fluor, N.A. = 0.5, Nikon) objectives. The 4 × objective was used to visualize the entire EPN containing the surrounding areas in a single frame in CLSM, whereas the 10 × and 20 × objectives were used to identify and analyze EPN neurons with sufficient resolution. The size of each frame was 1024 × 1024 pixels, and images of the optical slices were acquired from the section surface to the bottom at the preset optimal step size and were stored as a stacked file for each frame using the three single images for different fluorescence signals. The intensity of the signal in each pixel was recorded at 8 bits for each channel.

### Analysis

The extent of the EPN was determined based on the combination of the immunoreactivities for SP and NeuN. In the set of triple immunostaining that was not processed for this combination, the contour of the EPN obtained in adjacent sections that were double-stained for SP and NeuN was adapted. For the quantitative analysis of the distribution of neurons inside the EPN, every other section was chosen from the serial sections covering the entire EPN (three animals), and the CLSM images were acquired with a × 10 and × 20 objective. Quantitative data for ChAT-positive neurons were used from a previous study, because they are a minor population (~ 3.8%) of EPN neurons in mice (Miyamoto and Fukuda 2015). The numbers of NOS-, NOS/SOM-double, SOM-, PV-, and PV/NOS double-positive somata that were localized inside the EPN were quantified in each section using the computer-assisted neuron tracing system Neurolucida (MicroBrightField). According to the principle of the unbiased stereological disector method (Sterio 1984), the somata that were exposed to the top surface of each section, which were identified by examining serial optical slices, were excluded from the quantification. The number of cells quantified was averaged among the three animals at different positions along the rostrocaudal axis. The distribution was also represented as the numerical density per unit volume. The area of EPN in each section was measured by tracing the contour of the EPN with Neurolucida, and the numerical density was obtained by dividing the number of cells by the measured area and further by the section thickness. The total numbers of NOS-, NOS/SOM-double, SOM-, PV-, and PV/NOS double-positive neurons in the EPN were estimated by the sum of the number of cells quantified in every other coronal section along the rostrocaudal extent of the EPN and further by multiplying the sum by 2.

Quantitative analysis of neurons in FG-injected animals was performed in three sections per animal (three animals in total) corresponding to rostral (− 1.04 mm from bregma), middle (− 1.34 mm), and caudal (− 1.64 mm) parts of the EPN. The number of EPN neurons coexpressing FG in each section was counted.

## Results

### Characterization of the anti-NOS antibody

**Fig. 1:**
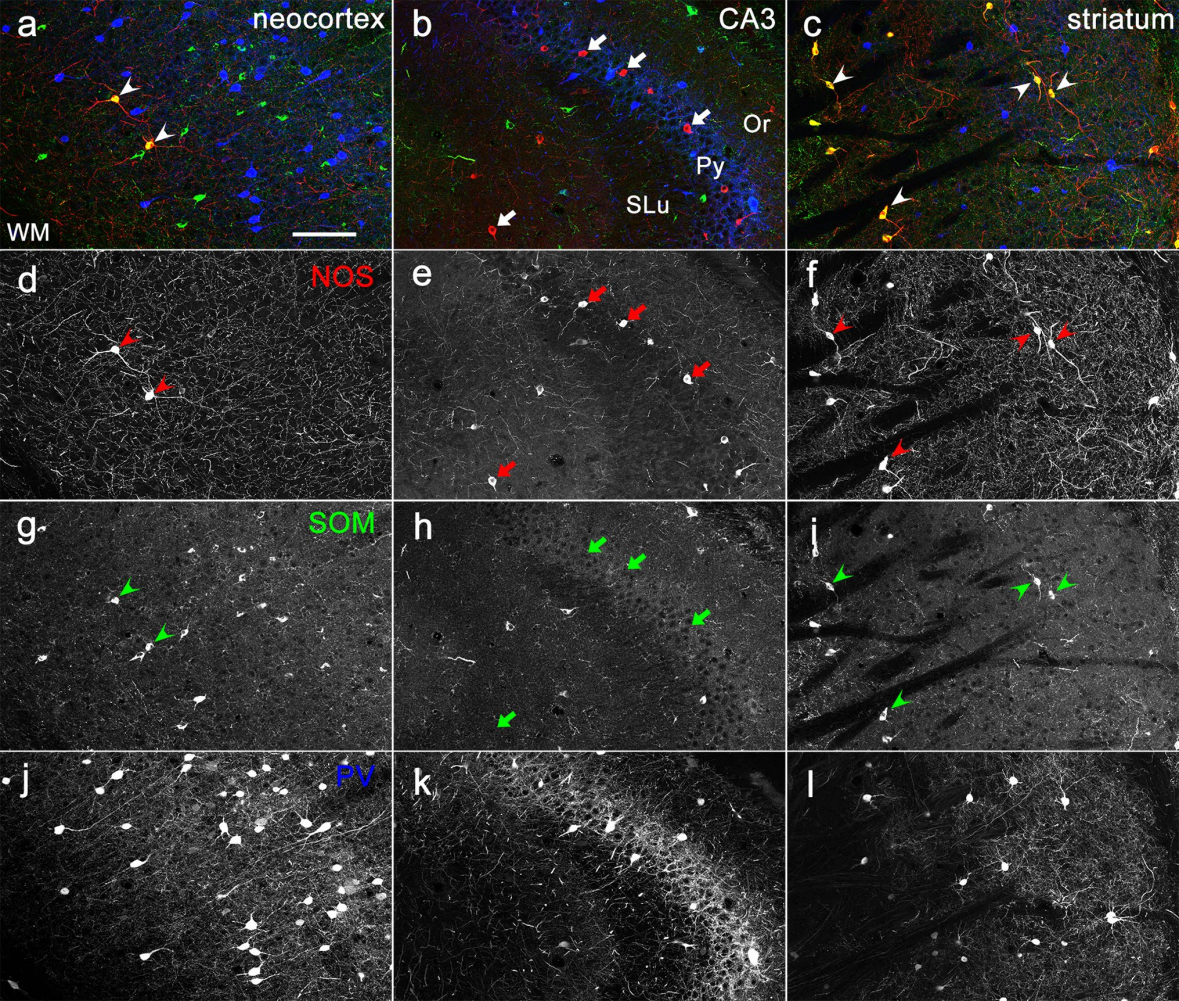
NOS immunoreactivity in three different brain regions. Each section showing the neocortex (**a**, **d**, **g**, **j**), CA3 region of the hippocampus (**b**, **e**, **h**, **k**) and striatum (**c**, **f**, **i**, **l**) was obtained from the same animal. Pseudo-color images in (**a**–**c**), consisting of immunolabeling for NOS (red), SOM (green), and PV (blue), are shown separately in (**d**–**f**), (**g**–**i**), (**j**–**l**), respectively. Arrows and arrowheads indicate NOS-positive and NOS/SOM-double positive neurons, respectively. Or, stratum oriens; Py, stratum pyramidale; SLu, stratum lucidum; WM, white matter. Scale bar = 100 µm

The anti-NOS antibody used in the present study is the classical one, the quality of which has been proven to be highly specific by both western blotting (Herbison et al. 1996) and immunostaining (Herbison et al. 1996; Jinno et al. 1999; Lin and Talman 2001; Lin et al. 2007; Ishihara and Fukuda, 2016). We confirmed the specific immunoreactivity with this antibody in several brain regions where pattern of NOS immunoreactivity has been established (Fig. 1). Both the morphological features of labeled neurons and the co-localization relationships with other neurochemical markers were consistent with findings in previous studies (Vincent et al. 1983; Kawaguchi et al. 1995; Dun et al. 1994a, 1994b). For example, NOS immunoreactivity in the neocortex was detected in large-sized, non-pyramidal neurons that were also labeled for SOM but not for PV (Fig. 1a, d, g, j). Intense labeling in numerous axons and axon terminals running throughout the cortical tissue was another characteristic feature of NOS immunoreactivity in the neocortex (Fig. 1d). In the hippocampus proper, many small-sized non-pyramidal cells were labeled for NOS, and the majority of them lacked immunoreactivity for both SOM and PV (Fig. 1b, e, h, k). The NOS immunoreactivity in the striatum was similar to that in the neocortex: non-spiny neurons of medium-to-large size were co-labeled for NOS and SOM but not for PV (Fig. 1c, f, I, l), with the occurrence of numerous labeled axons distributing diffusely in the striatum (Fig. 1f). All these results permitted us to apply this antibody safely to the EPN where immunoreactivity for NOS has not been fully analyzed.

### Identification of NOS-positive neurons in the EPN

**Fig. 2:**
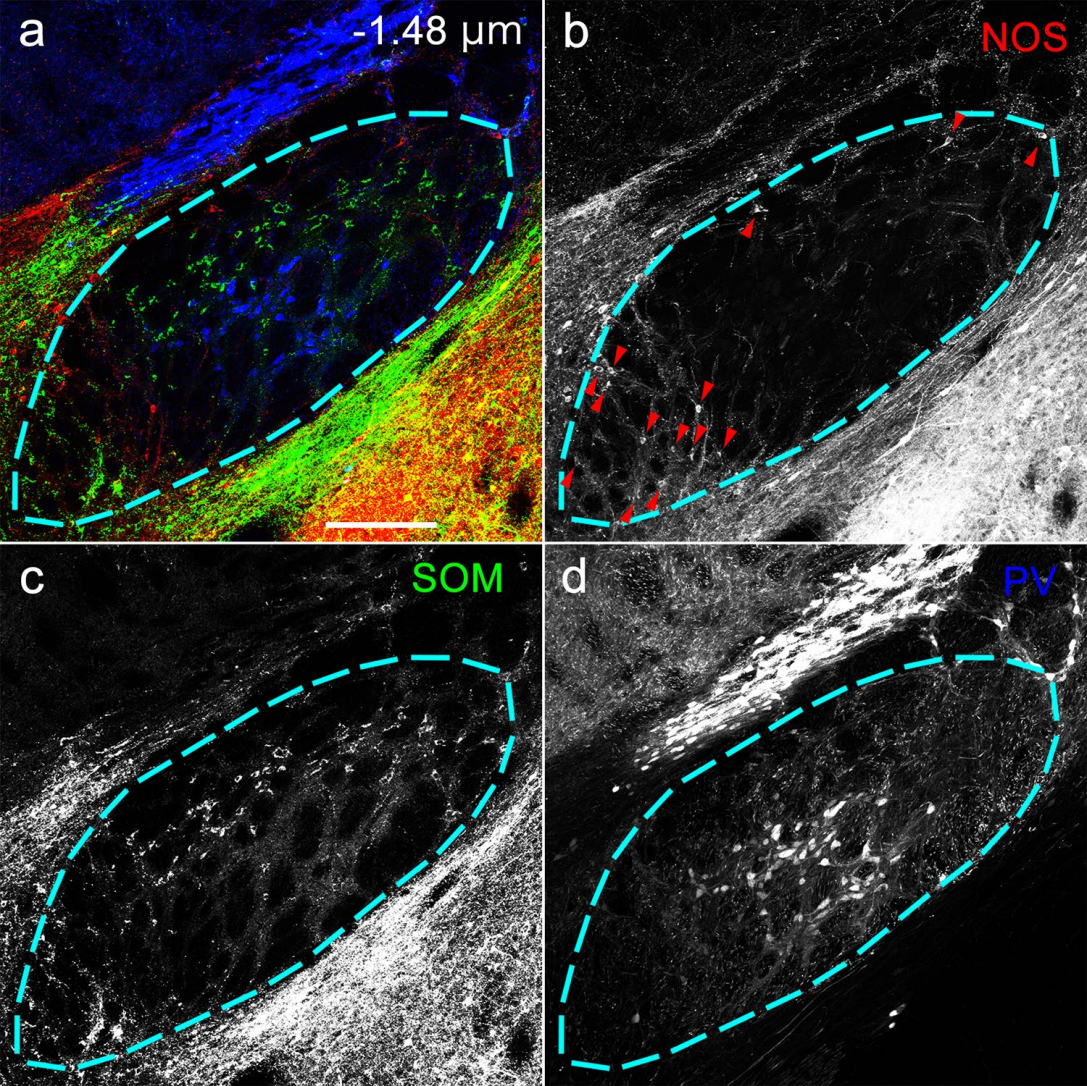
Triple-labeled immunohistochemistry for NOS, SOM, and PV in the caudal EPN. The pseudocolor image in (**a**) consists of NOS (red), SOM (green), and PV (blue) immunoreactivities, which are presented separately in (**b**–**d**). The area surrounded by the dashed line indicates the range of EPN determined by the SP immunoreactivity in the neighboring section. Arrowheads in (**b**) indicate NOS-positive neurons. The number in (**a**) represents the distance from bregma. Scale bar = 200 µm

**Fig. 3:**
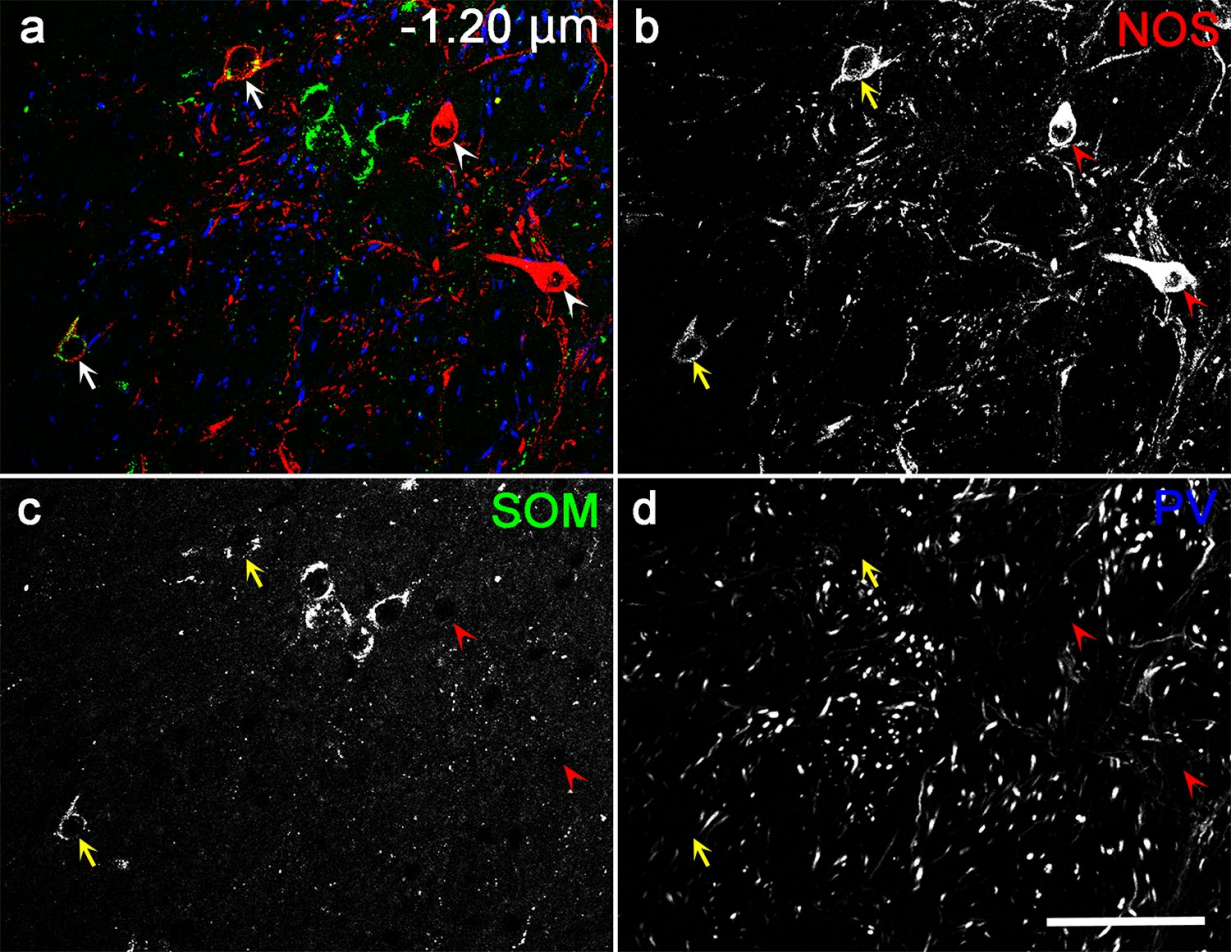
Triple-labeled immunohistochemistry for NOS, SOM, and PV in the rostral EPN. The pseudocolor image in (**a**) consists of NOS (red), SOM (green), and PV (blue) immunoreactivities, which are presented separately in (**b**–**d**). Arrows and arrowheads indicate NOS/SOM double-positive and NOS-only positive neurons, respectively. The number in (**a**) represents the distance from bregma. Scale bar = 50 µm

The EPN is known to consist of three different neuronal subpopulations that are immunoreactive for PV, SOM, or ChAT. However, we have previously reported the presence of another neuronal type that does not show immunoreactivity for any of these substances in the mouse EPN (Miyamoto and Fukuda 2015). To elucidate the immunohistochemical properties of these neurons, we processed every other coronal section for the two sets of triple immunohistochemistry using a mixture of antibodies against PV, SOM, and NOS (Figs. 2, 3) or another mixture of antibodies against SP, NeuN, and NOS. The immunoreactivity for SP was used to identify the extent of the EPN (Miyamoto and Fukuda 2015), because SP is localized in axon terminals that originate from the striatal direct pathway neurons targeting the EPN (Gerfen and Young 1988). The SP-defined extent of the EPN was also applied to the adjacent, paired sections. In the first set of triple labeling, neurons showing NOS immunoreactivity were observed in addition to the known populations showing immunoreactivity for either PV or SOM (Fig. 2). The majority of NOS-positive neurons lacked immunoreactivity for both PV and SOM, but some colocalized SOM, while NOS/PV double-positive neurons were rarely identified (Fig. 3). NOS-containing neurons were also distributed in the regions surrounding the EPN, such as the globus pallidus, lateral hypothalamus, and central amygdala.

### Distribution pattern of EPN neurons and quantitative analysis

**Fig. 4:**
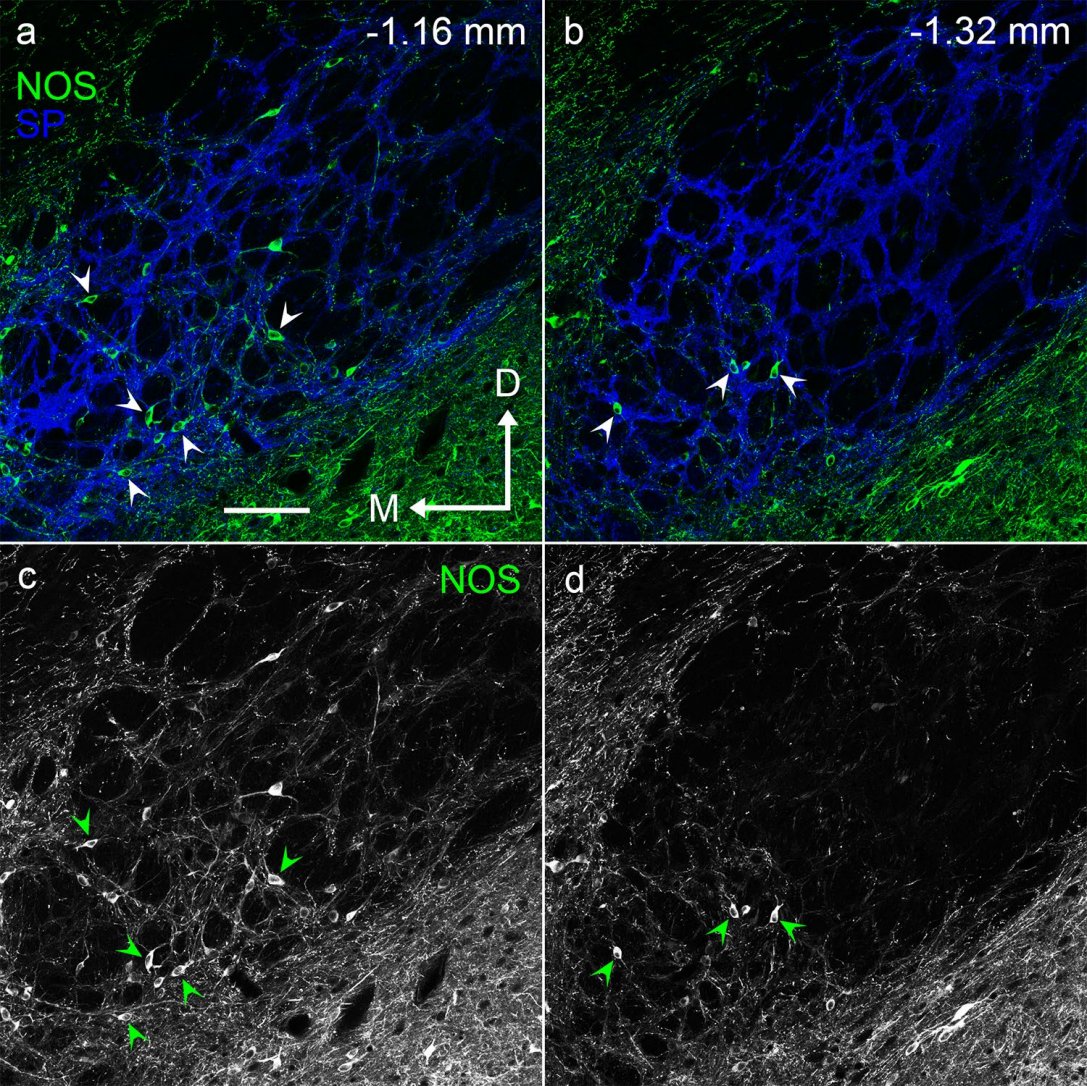
Localization of NOS-immunoreactive neurons at two different positions in the EPN. Pseudocolor images in (**a**, **b**) consist of double immunolabeling for NOS (green) and SP (blue), of which NOS immunoreactivity is shown in (**c**, **d**). NOS-positive neurons in the ventromedial part of the rostral and middle EPN (1.16 and 1.32 mm caudal to bregma) are shown by arrowheads. M = medial, D = dorsal. Scale bar = 100 µm.

**Fig. 5:**
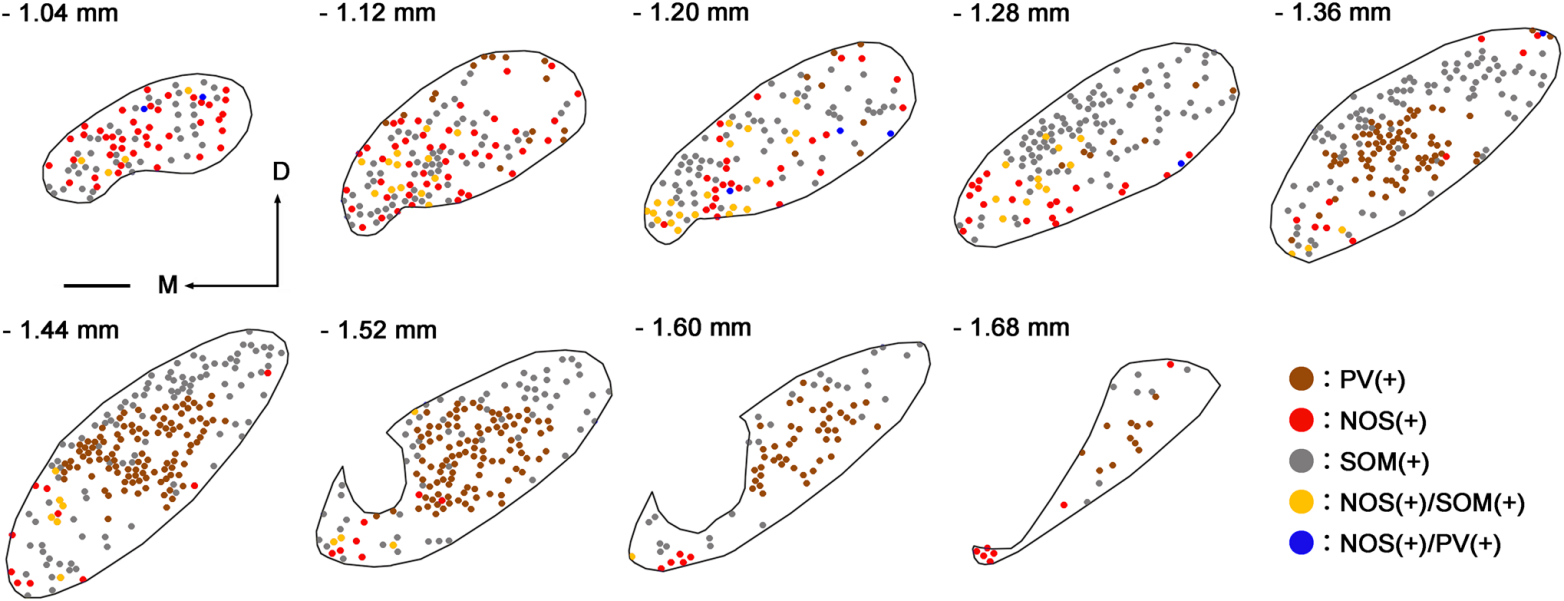
Distribution of five types of EPN neurons at the different rostrocaudal levels. PV-only, NOS-only, SOM-only, NOS/SOM-double, and NOS/PV double-positive neurons are indicated by dots of different colors. The number at the upper left of each panel shows the position relative to bregma. M = Medial, D = Dorsal. Scale bar = 200 µm

**Fig. 6:**
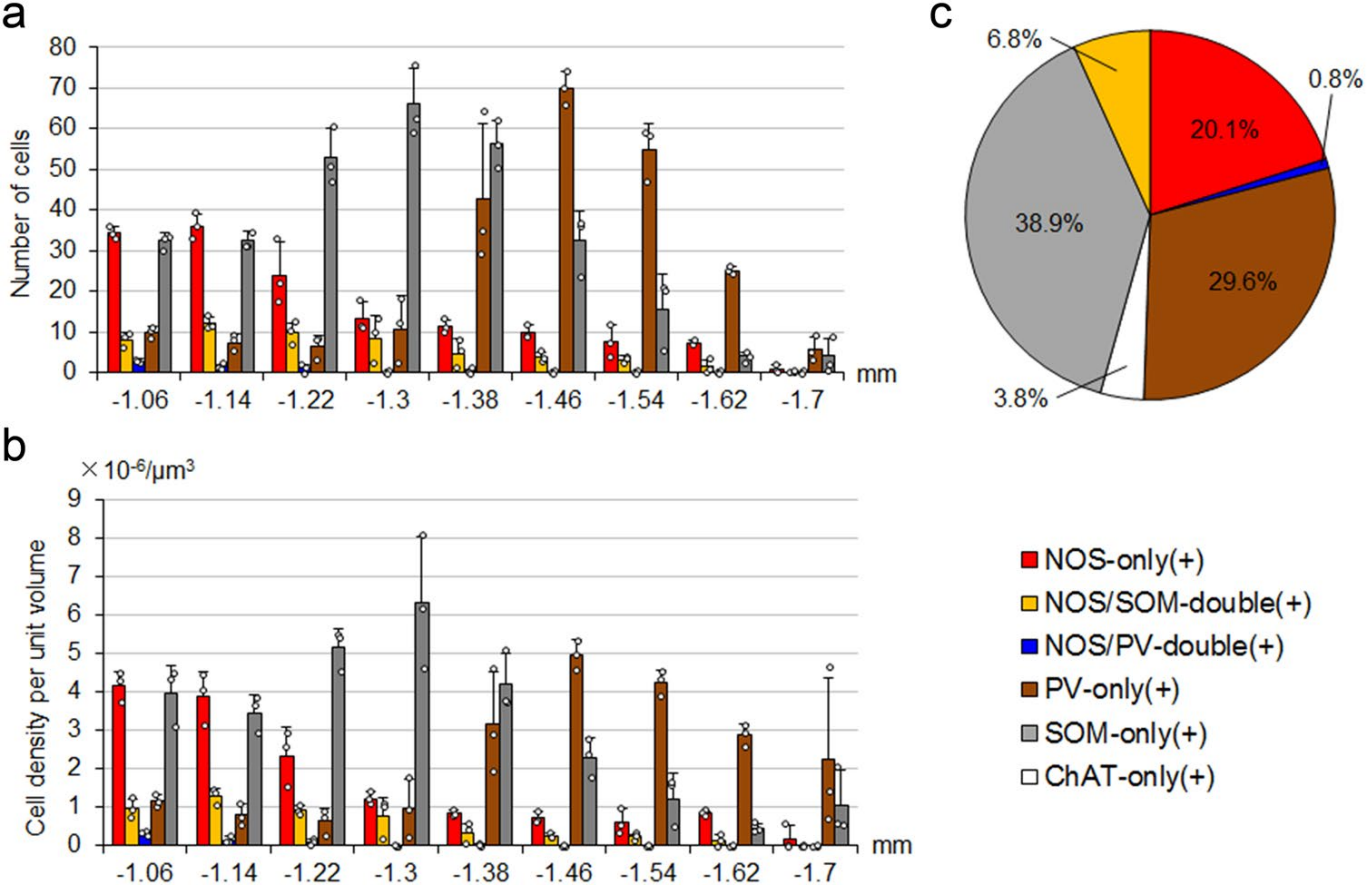
Quantitative analysis of EPN neurons. **a**, **b** The distributions of five types of neurons along the rostrocaudal axis in the EPN. The abscissa shows the relative position to bregma (mm). The ordinates in (**a**, **b**) show the number of cells in each section and the cell density per unit volume (mean ± standard deviation, *n* = 3 animals), respectively. Each dot represents a measured value obtained from a single section in each mouse. **c** Estimation of the proportion of the number of each type to all neuron number in the EPN

NOS-positive neurons were predominantly located in the rostral part of the EPN, and their number decreased caudally (Figs. 4, 6). NOS-positive neurons were scattered throughout the EPN in most rostral sections (− 1.04 and − 1.12 mm in Fig. 5). At the middle and caudal sections, their distribution gradually became localized to the ventromedial part of the EPN. As shown in our previous study (Miyamoto and Fukuda 2015), PV-positive neurons and SOM-positive neurons were distributed in complementary patterns in the EPN (Figs. 2, 5). PV-positive neurons occupied the central region of the EPN from the middle to the caudal parts along the rostrocaudal axis. In contrast, SOM-positive neurons surrounded the PV-positive core region in the caudal two-thirds of the EPN. In the rostral third of the EPN, SOM-positive neurons were widely distributed and intermingled with NOS-positive neurons.

The distribution patterns of the five types of neurons that were positive for NOS only, both NOS and SOM, both NOS and PV, PV only, and SOM only were analyzed quantitatively by disector, an unbiased stereological method (Sterio 1984; Miyamoto and Fukuda 2015). Figure 6 shows the number (a) and cell density (b; number per unit volume) of the five types in single sections located at different positions along the rostrocaudal axis. At the most rostral positions (− 1.06 and − 1.14 mm from bregma), neurons containing NOS and/or SOM accounted for the majority of EPN neurons. NOS-only positive neurons decreased toward the caudal part, whereas SOM-only positive neurons showed an increase toward the middle part and then gradually decreased caudally. PV-positive neurons were mainly located in the caudal two-thirds of the EPN, corresponding to the PV-rich core region (Figs. 2, 5). This multiplex distribution pattern indicates that EPN cannot be simply divided into rostral and caudal halves, as suggested in previous studies.

The proportions of PV-only positive, SOM-only positive, NOS/SOM double-positive, NOS-only positive, and NOS/PV double-positive neurons were 29.6 ± 4.0%, 38.9 ± 2.0%, 6.8 ± 1.3%, 20.1 ± 1.1%, and 0.8 ± 0.1%, respectively (Fig. 6c; *n* = 3 animals; sample size = 2186 cells). When these values were combined to obtain the proportions of PV- and SOM-positive neurons irrespective of the colocalization relationship, the values for PV- and SOM-positive neurons were 30.4% and 45.7%, respectively, which are in good correspondence to the proportions found in our previous study (PV, 28.6%; SOM, 45.7%; Miyamoto and Fukuda 2015). Moreover, the percentage of NOS-only positive neurons (20.1%) in the present analysis is comparable to the proportion of PV/SOM/ChAT-triple negative neurons (22.1%, Miyamoto and Fukuda 2015). This strongly suggests that PV/SOM/ChAT-triple negative neurons found in the previous study mostly correspond to NOS-only positive neurons. The proportion of neurons that did not show immunoreactivities for PV, SOM and NOS was 3.8%, which also corresponds well to the proportion of cholinergic neurons (3.6%) in our previous study (Miyamoto and Fukuda 2015). All these data led to the finding that the EPN consists of five different subpopulations, PV-only positive, SOM-only positive, NOS-only positive, NOS/SOM double-positive, and ChAT-positive neurons (six subpopulations if NOS/PV double-positive neurons are included), as shown in Fig. 6c.

### Projection targets of NOS-positive neurons

**Fig. 7:**
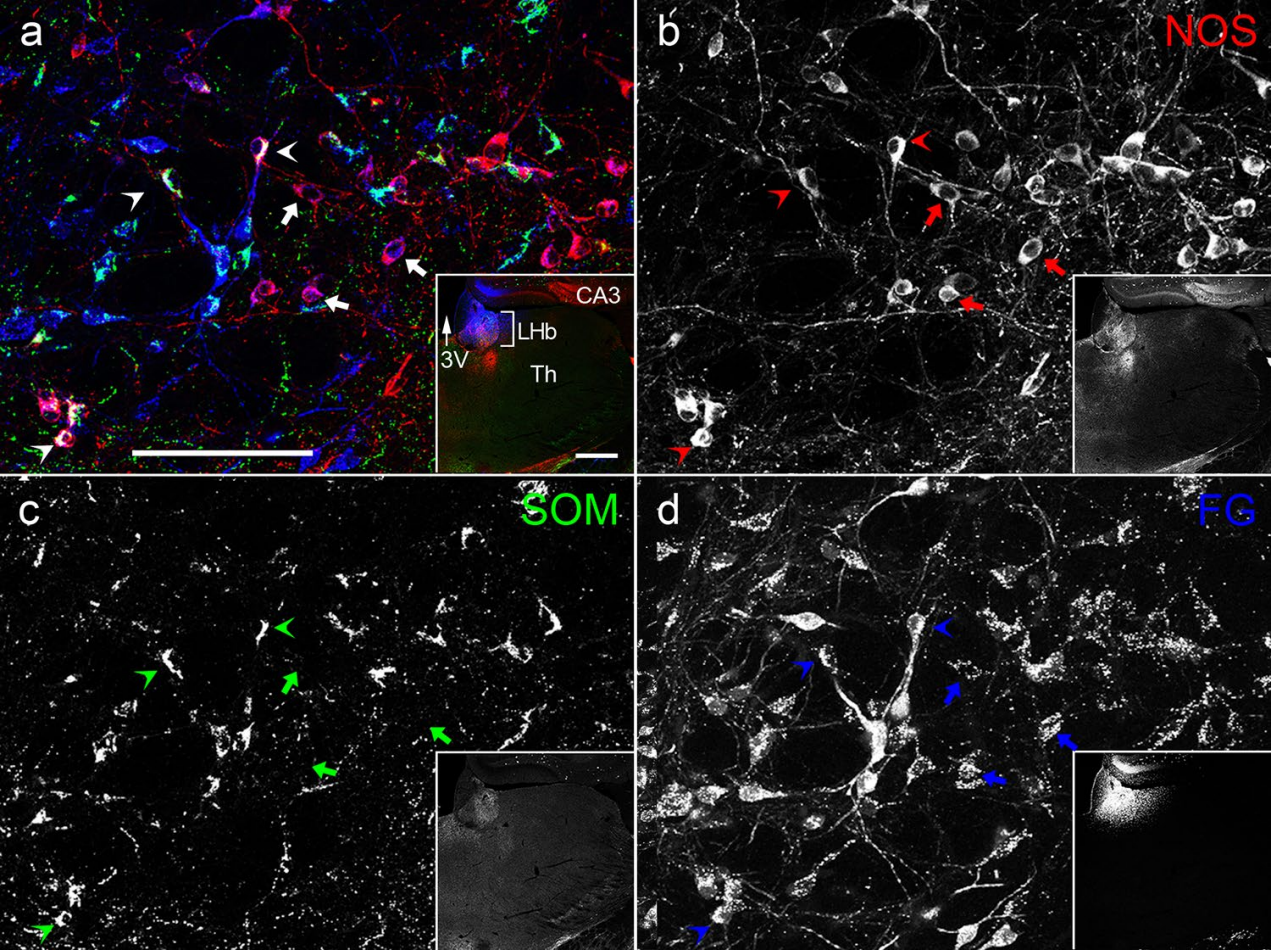
The colocalization relationship between retrogradely labeled FG, which was injected into the LHb, and NOS and/or SOM in the EPN. The pseudocolor images in (**a**) and Inset consist of NOS (red), SOM (green), and FG (blue) immunoreactivities, which are shown separately in (**b**–**d**). NOS/FG double-positive neurons and NOS/SOM/FG triple-positive neurons are shown by arrows and arrowheads, respectively. 3V, third ventricle; CA3, CA3 region of the hippocampus proper; Th, Thalamus. The scale bars in (**a**) and Inset are 100 and 500 µm

To determine the target areas of NOS-positive neurons, the retrograde tracer FG was injected into the LHb, two sites (rostral and caudal) of VA-VL, and FrA. Spread of FG in the LHb covered the whole nucleus (Fig. 7d Inset), and a large number of retrogradely labeled neurons were identified in the EPN (Fig. 7). These neurons consisted of SOM-, NOS-, or NOS/SOM double-positive neurons. In contrast, neither PV/FG double-positive nor ChAT/FG double-positive neurons were observed. The proportions of NOS-only positive, NOS/SOM double-positive, and SOM-only positive neurons among all FG-labeled neurons after injection into the LHb were 25.7 ± 2.2%, 15.2 ± 5.2%, and 59.1 ± 7.3%, respectively (Fig. 9a; *n* = 3 animals; sample size = 823 cells).

**Fig. 8:**
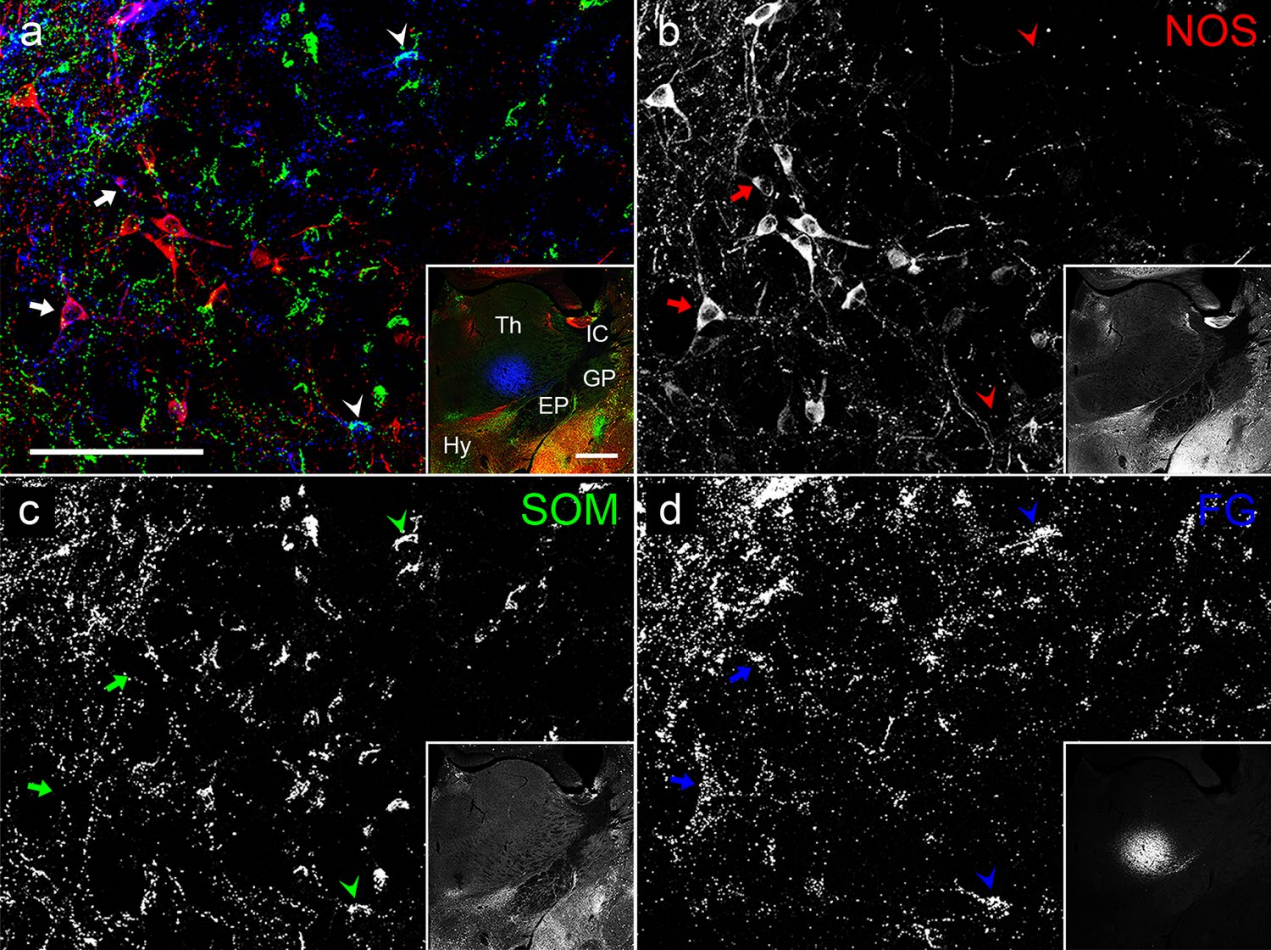
The colocalization relationship between the retrogradely labeled FG, which was injected into the VA-VL, and NOS and/or SOM in the EPN. The pseudocolor images in (**a**) and Inset consist of NOS (red), SOM (green), and FG (Blue) immunoreactivities, which are shown separately in (**b**–**d**). NOS/FG double-positive neurons are shown by arrows, whereas SOM/FG double-positive neurons are shown by arrowheads. EP, entopeduncular nucleus; GP, globus pallidus; Hy, hypothalamus; IC, internal capsule; Th, thalamus. The scale bars for images (**a**) and Inset are 100 and 500 µm, respectively

**Fig. 9:**
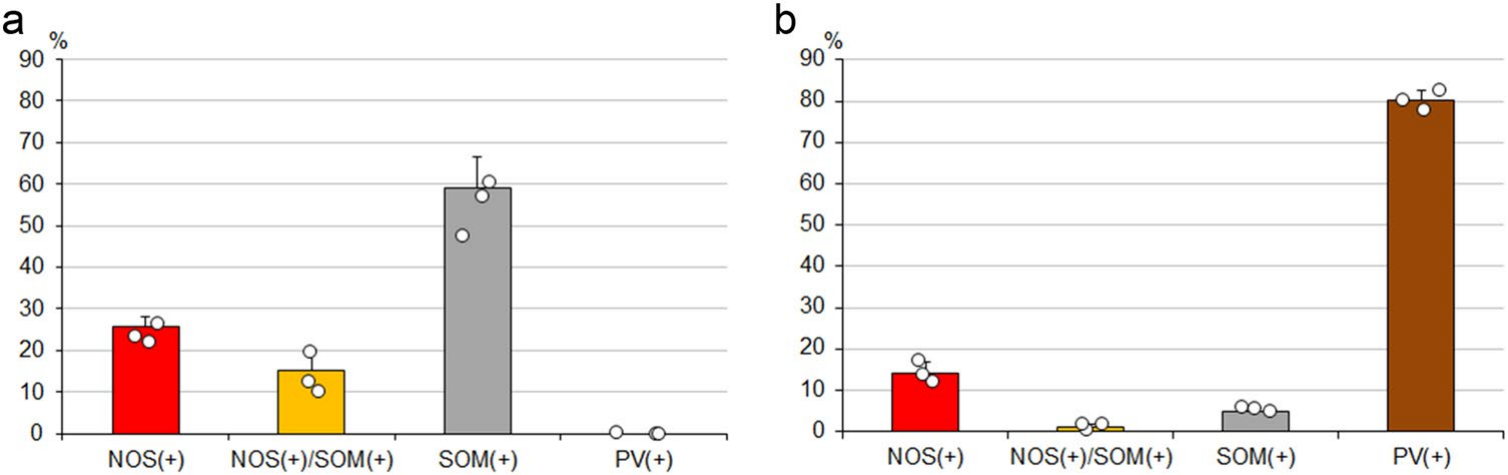
Analysis of the proportion of retrogradely labeled EPN neurons (mean ± standard deviation, *n* = 3 animals) after injections of FG into the LHb (**a**) and the VA-VL (**b**), classified by immunohistochemical markers. Each dot represents the proportion obtained from a single mouse

Next, we injected FG into the VA-VL thalamic nuclei, which are known as the main target of PV-containing neurons in the EPN. Two different sites along the rostrocaudal axis were selected for the injection. Spread of FG at the rostral and caudal injection sites did not overlap, but the injections at the two sites resulted in the same labeling pattern in the EPN. Thus, we analyzed the data collectively. Spread of FG in the VA-VL (Fig. 8d Inset) was comparable to that in the LHb (Fig. 7d Inset), but FG covered only a limited space of the nuclei, because the size of the VA-VL as a whole was much larger than that of the LHb. Although a number of PV-positive neurons were retrogradely labeled by FG, some NOS-positive and a few SOM-positive neurons also showed immunoreactivity for FG (Fig. 8). ChAT/FG double-positive neurons were not observed in these sections. The proportions of NOS-only positive, NOS/SOM double-positive, SOM-only positive, and PV-only positive neurons among all FG-labeled neurons after injection into the VA-VL nuclei were 14.0 ± 2.8%, 0.9 ± 0.8%, 5.0 ± 0.6%, and 80.1 ± 2.5%, respectively (Fig. 9b; *n* = 3 animals, 2 for rostral injection and 1 for caudal injection; sample size = 238 cells).

In the sections obtained from animals injected with FG in the FrA, a few retrogradely labeled neurons were identified in the EPN, and they showed immunoreactivity for ChAT but were not double-labeled with any other subpopulations (data not shown).

**Fig. 10:**
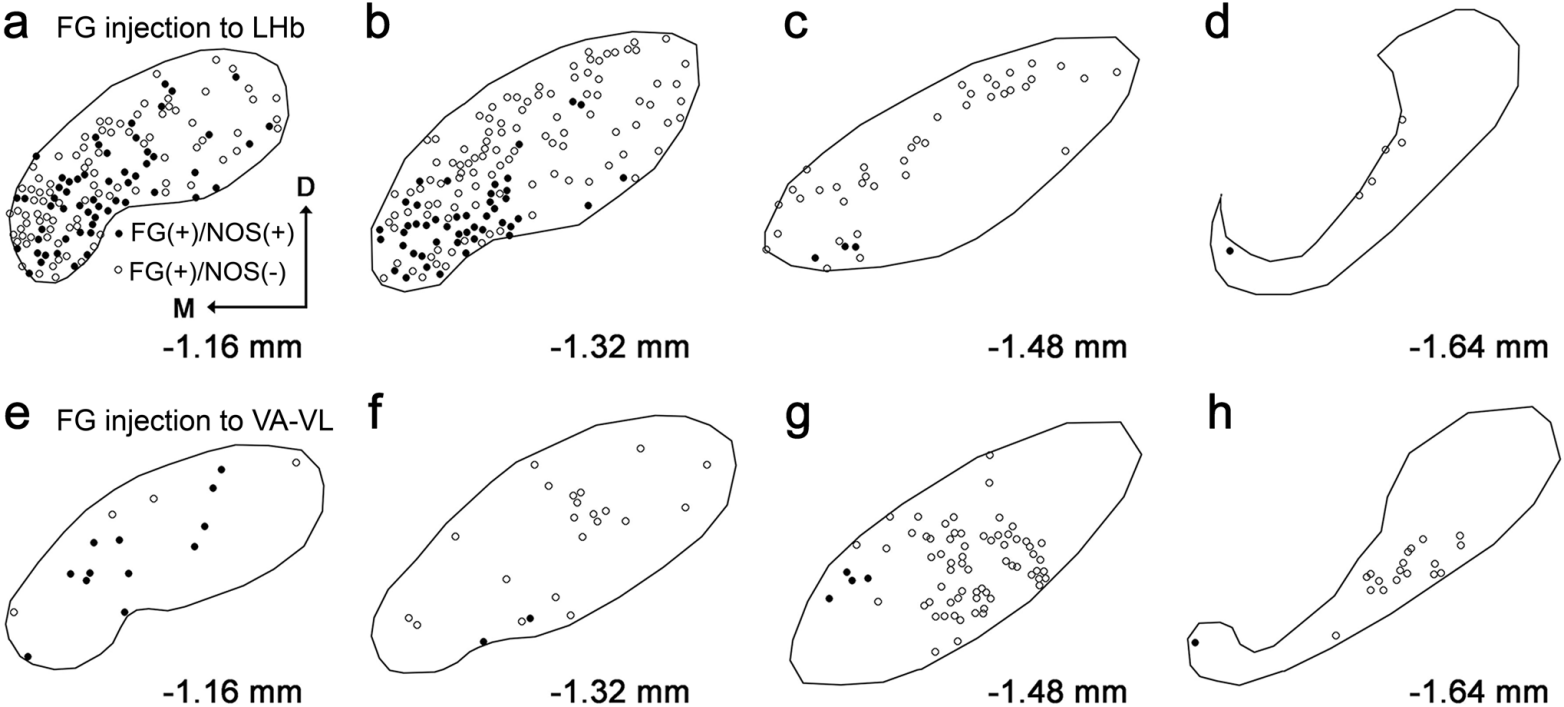
Distribution of EPN neurons labeled by FG injection into the LHb (**a–d**) and VA-VL (**e–h**). Each solid and open circle represents the position of NOS( +)/FG( +) and NOS(-)/FG( +) neuron, respectively. The position along the rostrocaudal axis is shown as the distance from bregma. Note that the number of labeled cells cannot be compared directly between the two experiments because of the difference in relative spread of FG in the injection site

Finally, the distribution of NOS-positive neurons that were retrogradely labeled after FG injection was examined. In sections obtained from mice injected with FG into the LHb (Fig. 10a–d), NOS/FG double-positive neurons were mainly localized in the rostral and ventromedial parts of the EPN, which was consistent with the distribution of NOS-containing neurons described above. Large numbers of NOS-negative/FG-positive neurons were also observed in the same experiments. Most of these neurons are thought to correspond to SOM-positive neurons, because their distribution was relatively homogeneous in the rostral part but was limited to the peripheral shell region in the middle part of the EPN.

Regarding the distribution of FG-labeled neurons after injection into the VA-VL (Fig. 10e–h), a small number of NOS/FG double-positive neurons were observed in the rostral part of the EPN. In the same experiments, a large number of NOS-negative/FG-positive neurons were further identified in the middle and caudal parts of the EPN. These neurons showed a core-like distribution pattern, which corresponded well to the distribution of PV neurons. These findings further demonstrate that the two populations of NOS neurons projecting to the different targets, LHb and VA-VL, intermingled with each other in the rostral part of the EPN, although it cannot be determined whether axon collaterals toward the LHb and VA-VL originate from the same neurons.

## Discussion

The present study revealed the unidentified neuronal population in the mouse EPN and examined their projection targets. The main findings are summarized as follows:

1. A group of neurons showing immunoreactivity for NOS constitutes the third major population of EPN neurons.
2. NOS-only positive and NOS/SOM double-positive neurons account for 20.1% and 6.5% of all EPN neurons, respectively.
3. NOS-immunoreactive neurons are distributed predominantly in the rostral half of the EPN, where they are mainly located in the ventromedial area.
4. NOS-immunoreactive neurons project primarily to the LHb, and some also project to the VA-VL.

### Neurochemical characterization of NOS-only positive neurons

Our previous analysis of EPN neurons revealed the presence of SOM/PV/ChAT-triple negative neurons (Miyamoto and Fukuda 2015). The results of the present study yielded the conclusion that this population mostly corresponds to NOS-only positive neurons. Because the SOM/PV/ChAT-triple negative population consists of glutamic acid decarboxylase (GAD)-positive neurons (14.4%) and GAD-negative, presumptive glutamatergic neurons (7.7%) (Miyamoto and Fukuda 2015), it seems reasonable to deduce that NOS-only positive neurons can be divided into GAD-positive and negative populations. In fact this was confirmed in our unpublished observations in colchicine-pretreated animals, in which both GAD-positive and negative subpopulations were identified in NOS neurons of the EPN under the experimental conditions where colchicine successfully induced intrinsically weak GAD-immunoreactivity in somata of striatal medium spiny neurons (Ribak et al. 1979). Therefore, excitatory transmission in the EPN–LHb pathway (Shabel et al. 2012) will be mediated by not only SOM neurons (Wallace et al. 2017) but also the presumptive glutamatergic subpopulation of NOS neurons. It remains unknown whether GAD-positive NOS neurons release both GABA and glutamate as observed in SOM-positive EPN neurons (Shabel et al., 2014; Wallace 2017).

### The internal division of the EPN

The EPN has been divided into rostral and caudal halves depending on the type of neurons and their projection targets (van der Kooy and Carter 1981; Rajakumar et al. 1994). However, our recent study revealed that the EPN can be viewed as a double elliptical structure, which we termed core/shell organization, based on the immunohistochemical properties (Miyamoto and Fukuda 2015). The core region occupies the center of the caudal EPN and is enriched with PV neurons, whereas PV-negative neurons surround the PV-rich core in the caudal EPN and further extend rostrally to occupy the whole body of the rostral EPN. The present results show that not only SOM neurons but also NOS neurons occupy this shell-like zone at both the rostral and caudal levels. Thus, the position of the core that corresponds to the internal ellipsoid deviates caudally inside the external ellipsoid that represents the whole EPN. The tracer experiments further substantiate the core/shell organization in that the distributions of the thalamus- and lateral habenula-targeting neurons correspond well to the core and shell zones, respectively. From this perspective, NOS neurons can be characterized as shell neurons targeting mostly the lateral habenula. Similar structures are mentioned as “core” and “periphery” organization in the primate GPi (Parent and Bellefeuille 1982).

Although SOM neurons are distributed diffusely in the shell, NOS-immunoreactive neurons are mainly located in the ventromedial part of the shell. This suggests that the shell, defined by both the accumulation of lateral habenula-targeting neurons and the enrichment of PV-negative neurons, contains an NOS-rich subarea.

### Projection targets of NOS-immunoreactive neurons

From past studies, the projection targets of EPN neurons can be related to the differences in the chemical characteristics of the neurons. EPN neurons projecting to the thalamus contain PV (Rajakumar et al. 1994), whereas the projection target of the SOM-containing EPN neurons is the LHb (Vincent and Brown 1986). The localization patterns of these two neuronal types coincide well with the core/shell organizations (Miyamoto and Fukuda 2015). However, this dichotomous view needs to be updated, because the present results demonstrate a new population, NOS positive neurons, in the EPN. NOS neurons were distributed in the EPN shell and projected to the LHb just as SOM-positive neurons. Moreover, a certain number of NOS-immunoreactive neurons were labeled after FG injection into the VA-VL, and these neurons were located also in the shell. This suggests two possibilities: distinct subpopulations of NOS neurons target either LHb or VA-VL but are located in the same division of the EPN, or single NOS neurons send axon collaterals to both LHb and VA-VL. The present results did not solve this issue, and previous studies regarding the connectivity between the EPN and its target regions led to controversial results. One study reported that distinct types of EPN neurons projected to the thalamus and LHb (Rajakumar et al. 1993), whereas another study showed that some EPN neurons projecting to the LHb extended the axon collateral to the thalamus (Kha et al. 2000). In relation to these two possibilities, another point to be considered is the relative abundance of the retrogradely labeled cells. Because the relative coverage of the injection site by FG was much smaller in the VA-VL as compared to the full coverage in the LHb, the number of labeled EPN cells after a single injection to the VA-VL as shown in Fig. 10 is thought to be an underestimate. Thus, apparently fewer labeling after the VA-VL injection does not necessarily exclude the possibility that many LHb-targeting neurons have collateral axons to the thalamus. Future studies are needed to resolve this important issue, because the EPN plays a pivotal role in sending information processed inside the basal ganglia toward many brain regions to execute appropriate behaviors.

### Functional implications

LHb-projecting neurons in the primate GPi respond to both nonreward and reward cues, with their activities increasing and decreasing after cue presentation, respectively (Hong and Hikosaka 2008, 2013). In the rodent EPN, LHb-projecting GP (GPh) neurons are important in controlling the valence and responses to aversive stimuli (Shabel et al. 2012; Stephenson-Jones et al. 2016). A recent study using in vivo electrophysiology showed that individual GPh neurons located in the EPN are classified into two types, inhibitory (reward cue-inhibited neurons) and excitatory (reward cue-excited neurons), by presentation of reward cues, and that each type contrastingly responds toward aversive stimuli in a direction opposite to that toward reward cues (Li et al. 2019). Furthermore, LHb neurons of different subpopulations also take a response pattern similar to either of the two patterns in EPN neurons (Li et al. 2019), suggesting the possibility that EPN–LHb pathway encoding valence consists of parallel projections. However, this scheme is complicated by the co-release of GABA/glutamate from axon terminals of SOM-positive presynaptic neurons onto postsynaptic LHb neurons (Shabel et al. 2012, 2014; Wallace 2017). It has been demonstrated that the dual antagonistic responses in LHb neurons depend on the balance and synaptic strength between GABA and glutamate transmission (Li et al. 2011; Meye et al. 2016), which might be mediated through modulation of GABA/glutamate co-releasing synapses from single neurons. Alternative possibility is that the dual responses in the LHb can be mediated by afferent signals from distinct subsets of EPN neurons, one predominantly glutamatergic and the other GABAergic. Most of SOM-positive GPh neurons express both GAD and VGluT2, but they predominantly behave as excitatory neurons (Wallace et al. 2017). This brings up an issue of whether there is a subset of predominantly GABAergic EPN neurons. NOS-only positive neurons showing GAD immunoreactivity might be a candidate of GABAergic subset in the EPN-LHb circuitry.

A recent study using the single cell analysis of dissolved neurons from the EPN demonstrated the existence of purely glutamatergic GPh neurons that belong to a minor subpopulation of PV neurons (Wallace et al. 2017). These neurons were identified as PV neurons based on the results in both optogenetic manipulation of PV-Cre mice and in situ hybridization study to detect PV mRNA. However, our previous quantitative immunohistochemical analysis led to some different conclusion that all PV neurons in the EPN were GAD-positive (Miyamoto and Fukuda 2015). Moreover, in the present analysis, no PV-positive neurons were found among 823 FG-labeled EPN neurons after FG injection into the LHb (Fig. 9a). These discrepancies might be explained by the methodological difference between the mRNA-driven analysis and immunohistochemical detection of expressed protein. There are several examples of very low to almost no immunohistochemical signals in somata of neurons that should express the proteins, such as GAD in the medium-sized spiny neurons in the striatum (Ribak et al. 1979), and GAD65 in both hippocampal PV neurons (Fukuda et al. 1997) and cerebellar Purkinje cells (Esclapez et al. 1994; Obata et al. 1999). Thus, expression of PV protein in purely glutamatergic neurons of the EPN might be under the detection level in conventional immunohistochemical procedures.

Taken together, to interpret the diverse functional properties of LHb neurons in response to aversive stimuli, at least four separate groups of GPh neurons should be taken into consideration: SOM-positive neurons that co-release GABA and glutamate (Shabel et al. 2014; Wallace et al. 2017), purely glutamatergic PV neurons (Wallace et al. 2017), and NOS-positive neurons that consist of both GABAergic and glutamatergic subpopulations. The possibility of GABA/glutamate co-release from GABAergic NOS neurons needs to be determined in future studies.

NO, which is synthesized from L-arginine by the action of NOS, is used as a neurotransmitter, neuromodulator, and neural messenger in the central nervous system (Snyder and Bredt 1991). It acts diffusively on both anterograde and retrograde signals independent of synapses and receptors, because NO is a lipophilic and gaseous molecule (Brenman and Bredt 1997; Dawson and Snyder 1994). One of the important functions of NO is synaptic plasticity, such as long-term potentiation and depression (Calabresi et al. 1999; Hardingham et al. 2013; Lev-Ram et al. 1997; Schuman and Madison 1994). Since synaptic plasticity is a basic neural mechanism for learning and memory, NO might also be involved in several functions of the basal ganglia, such as the initiation of voluntary movements, control of procedural memory, and goal-directed action generation, leading to cognitive rewards. Therefore, NOS-containing neurons found in this study may be one of the important pieces to coordinate these activities.

## Data Availability

All data and materials are available on request.
